# Effects of a Nurse-Led Telehealth Self-care Promotion Program on the Quality of Life of Community-Dwelling Older Adults: Systematic Review and Meta-analysis

**DOI:** 10.2196/31912

**Published:** 2022-03-21

**Authors:** Arkers Kwan Ching Wong, Jonathan Bayuo, Frances Kam Yuet Wong, Wing Shan Yuen, Athena Yin Lam Lee, Pui King Chang, Jojo Tsz Chui Lai

**Affiliations:** 1 School of Nursing The Hong Kong Polytechnic University Kowloon Hong Kong

**Keywords:** telehealth, meta-analysis, self-care, community-dwelling older adult, nurse

## Abstract

**Background:**

In recent years, telehealth has become a common channel for health care professionals to use to promote health and provide distance care. COVID-19 has further fostered the widespread use of this new technology, which can improve access to care while protecting the community from exposure to infection by direct personal contact, and reduce the time and cost of traveling for both health care users and providers. This is especially true for community-dwelling older adults who have multiple chronic diseases and require frequent hospital visits. Nurses are globally recognized as health care professionals who provide effective community-based care to older adults, facilitating their desire to age in place. However, to date, it is unclear whether the use of telehealth can facilitate their work of promoting self-care to community-dwelling older adults.

**Objective:**

This review aims to summarize findings from randomized controlled trials on the effect of nurse-led telehealth self-care promotion programs compared with the usual on-site or face-to-face services on the quality of life (QoL), self-efficacy, depression, and hospital admissions among community-dwelling older adults.

**Methods:**

A search of 6 major databases was undertaken of relevant studies published from May 2011 to April 2021. Standardized mean differences (SMDs) and their 95% CIs were calculated from postintervention outcomes for continuous data, while the odds ratio was obtained for dichotomous data using the Mantel–Haenszel test.

**Results:**

From 1173 possible publications, 13 trials involving a total of 4097 participants were included in this meta-analysis. Compared with the control groups, the intervention groups of community-dwelling older adults significantly improved in overall QoL (SMD 0.12; 95% CI 0.03 to 0.20; *P*=.006; *I*^2^=21%), self-efficacy (SMD 0.19; 95% CI 0.08 to 0.30; *P*<.001; *I*^2^=0%), and depression level (SMD –0.22; 95% CI –0.36 to –0.08; *P*=.003; *I*^2^=89%).

**Conclusions:**

This meta-analysis suggests that employing telehealth in nurse-led self-care promotion programs may have a positive impact on older adults, although more studies are needed to strengthen the evidence base, particularly regarding organization and delivery.

**Trial Registration:**

PROSPERO (Prospective International Register of Systematic Reviews) CRD42021257299; https://www.crd.york.ac.uk/prospero/display_record.php?RecordID=257299

## Introduction

Aging populations put tremendous pressure on health and social care systems. Encouraging self-care practices and independent living among older adults has been regarded as one of the best solutions to reduce the demands on costly tertiary and institutional care services [1]. Older adults have the responsibility to make an effort to adopt positive personal health practices according to their own preferences. By adopting such self-care practices, they can maintain their autonomy and independence, and enjoy an improved quality of life (QoL) [2].

Nurses are believed to play the most prominent role in promoting self-care behaviors among older adults [3]. Numerous studies provide evidence of their competence and capability in relation to preventive interventions, including their use of comprehensive and systematic assessments that facilitate early identification of older adults’ health complaints [3], their adoption of a holistic caring approach that addresses multiple complaints [4], their capacity to make referrals to other health professionals in a multidisciplinary team if needed [5], and their ability to build a trusting relationship with older adults [6]. However, previous nurse-led self-care promotion interventions relied heavily on a supportive environment that allowed only for face-to-face communication, and so can be difficult to implement in the face of existing barriers in health care institutions, such as time constraints [7], and transportation issues for those with physical or functional limitations [8]. These obstacles can jeopardize the quality of the interventions and the eventual health outcomes and QoL of the older adults in need of care [9]. It is thus better to take those interventions to the community level, including patients’ homes, in the hope of obtaining sufficient time, geographical convenience, and greater familiarity and security for the introduction of these preventative measures. Although the new practice may also cost a considerable amount of time and manpower, using telehealth as a solution to delivering care may make possible the realization of this vision of “nurse-led preventive community care for all.”

Telehealth refers to the services that bring health care directly to users, generally in their own homes, supported by information and communication technology [10]. It includes but is not limited to social alarms, lifestyle monitoring, remote monitoring of vital signs for diagnosis, and long-distance assessment and education. With the assistance of telecommunication tools such as smartphones, audio or video equipment, or tablets, telehealth changes the geography of health care by introducing person-centered virtual communication contexts, such as videoconferencing, telephone calls, and SMS text messages [11,12]. The benefits of telehealth are evident because from a geographical perspective it enables care to be delivered at a distance and improves access to care under different conditions. For instance, health care providers are able to reach out to older adults who are socially isolated or physically homebound due to diseases, disabilities, or other family roles. It has also helped to minimize the risk of direct transmission of infectious diseases for both health care providers and older adults during the COVID-19 pandemic [13]. Meanwhile, from a psychosocial perspective, it redefines familiar places (eg, the homes of older adults) into spaces of care [11]. Without geographical restrictions and the associated concerns, both older adults and their health care providers can devote more time and attention on the interventions themselves, resulting in an improvement in the quality of care that is provided. Indeed, these benefits are in accordance with López’s [14] view that telehealth is a technological catalyst for the implementation of community-based aging-in-place care systems. It elevates both the access to and quality of nurse-led self-care promotion programs in the community, transforming them into unique and holistic preventative measures that effectively increase the QoL of community-dwelling older adults [15], as well as achieving the goal of relieving the burden on health systems.

Despite the apparent benefits of nurse-led telehealth programs on promoting self-care, reviews are lacking of its impact on the QoL of community-dwelling older adults and on health care systems. Previous reviews have mainly focused on the impact of such programs on caregivers instead of on the older adults themselves [9,16,17]. Some focused on patients with a specific disease or who were in the terminal phase of their life, instead of on a sample representing the general population of community-dwelling older adults [18-20], while others overlooked the leading efforts of nurses in using telecare to promote self-care in the community [21]. Little is therefore known about the effects of nurse-led telecare programs on promoting self-care among community-dwelling older adults.

This study is, to the best of our knowledge, the first systematic review and meta-analysis of randomized controlled trials (RCTs) aimed at summarizing evidence on the effects of nurse-led telehealth self-care programs on community-dwelling older adults compared with the usual on-site or face-to-face care. The particular focus is on the quality of the care that is delivered, as well as on other outcomes including self-efficacy, depression, and hospital admissions. Given the popularity of adopting and sustaining telehealth in promoting self-care during the COVID-19 pandemic and in the near future, the empirical evidence from this study may guide the efforts of policymakers to address challenges in providing services for this large but still overlooked segment of the population.

## Methods

### Overview

This systematic review and meta-analysis followed the Preferred Reporting Items for Systematic Reviews and Meta-analyses (PRISMA) statement.

### Search Strategy

Three investigators (PKC, WSY, and AYLL) independently conducted a literature search using CINAHL, MEDLINE (PubMed), EMBASE (Ovid), PsycINFO (BSCO), Web of Science, the Cochrane Central Register of Controlled Trials, and ClinicalTrials.gov to identify RCTs written in English and published between May 2011 and April 2021. Given the rapidly changing nature of technology and the major changes that have taken place in the field of health care within the past 10 years, the goal was to capture the newest and most relevant evidence related to the use of telehealth as a self-care promotion intervention for community-dwelling older adults. Any disagreements were resolved by consensus with a fourth author (AKCW).

The following search strategy was used: (telehealth OR telecare OR telemedicine OR gerontechnology OR eHealth OR mHealth OR “mobile health” OR telecommunication OR teleconsultation OR teleconference) AND (self-care OR self-help OR self-management OR “self care” OR “self help” OR “self management”) AND (home OR “home health” OR “home care” OR community) AND (elderly OR aged OR aging OR ageing OR old* OR “older adult*” OR senior OR geriatric OR “older person” OR “elderly person”) AND (nurs* OR nurse-led) AND (random* OR control* OR “usual care”). The online search was supplemented by an extensive hand search of the literature through references identified from retrieved articles. Gray literature such as abstracts, conference proceedings, and editorials was excluded.

### Study Selection

The criteria for inclusion in this meta-analysis were: (1) RCT; (2) conducted with adults aged 60 or over and living independently in the community; (3) using telehealth (defined as the use of apps, websites, WhatsApp, SMS text messages, email, social media such as Facebook or Twitter, telephone calls, tablets, software such as Zoom or Microsoft Teams, home remote monitoring devices [reactive or proactive], or any combination of these as a health care delivery channel) as an intervention group component; (4) using a face-to-face or on-site care service as the control group component; (5) intended to empower or promote the self-care of community-dwelling older adults (ie, self-care refers to an activity that individuals undertake on their own behalf to stay fit, maintain good health and functioning, and prevent illness, with or without assistance). Studies were excluded if (1) they focused on cognitively or functionally impaired older adults unable to perform self-care; and (2) they compared 1 or more telecare interventions without a comparison with a control group or with a no intervention control group. As this meta-analysis targeted interventions led by nurses, studies that included an interdisciplinary care team should have had nurses carry out at least 50% of the interventions.

For each article included in the review, data about the participants (country, number of participants, inclusion and exclusion criteria), interventions (components of both intervention and control groups, provider, duration), and outcomes (outcome measures, results) were extracted. These were then compared and analyzed. If the aforementioned data were not available, we contacted the corresponding researcher of the study in question to clarify and request missing information.

### Outcomes

The primary outcome of interest was QoL. Secondary outcomes of interest were self-efficacy, depression, and hospital admissions.

### Quality Assessment

The potential risk of bias in the included studies was evaluated using Cochrane Collaboration’s tool for assessing the risk of bias according to the Cochrane Handbook for Systematic Reviews of Interventions [22]. This tool was used to assess the quality of the included studies by monitoring 7 domains: random sequence generation, allocation concealment, the blinding of participants and personnel, the blinding of the outcome assessment, incomplete outcome data, selective reporting, and other biases [22]. Three authors (PKC, WSY, and AYLL) independently rated the studies according to the assessment tool. Disagreements were resolved through discussion with a fourth author (AKCW).

### Data Synthesis and Statistical Analysis

Meta-analyses were conducted using Review Manager (version 5.3). We performed a meta-analysis when a minimum of 2 studies compared the effects of an intervention over the treatment delivered to the control group at the longest follow-up time. Because of the foreseeable complexities and multicomponent nature of nurse-led self-care promotion programs, the research team decided to conduct a random-effects meta-analysis a priori. The accuracy of using this method was tested using a standard χ^2^ test and an inconsistency index (*I*^2^>50% or *P*<.05 or both). We planned to run a meta-regression using R (version i386 3.3.2; R Foundation) to explain the between-trial heterogeneity, but because fewer than 10 trials were included, such an approach was not possible [22]. The standardized mean differences (SMDs) and their 95% CIs were calculated from the postintervention outcomes for continuous data, while the odds ratio (ORs) was obtained for dichotomous data by using the Mantel–Haenszel test. The SMD effect sizes were considered small, moderate, and large when the value was <0.4, 0.4-0.7, and >0.7, respectively [22]. Pooled ORs (95% CI) were calculated and a 2-sided *P*-value <0.1 was adopted to indicate statistical significance [22]. Where a sensitivity analysis was required, the analysis was repeated but with the exclusion of studies with a low study quality/high risk of bias, or lacking a thorough explanation of the timeframe of the reported outcome, the study design, or participant characteristics. Publication bias was checked using a visual inspection of funnel plots [22] and calculated using the Egger bias test [23].

## Results

### Search Outcomes

**Figure 1:**
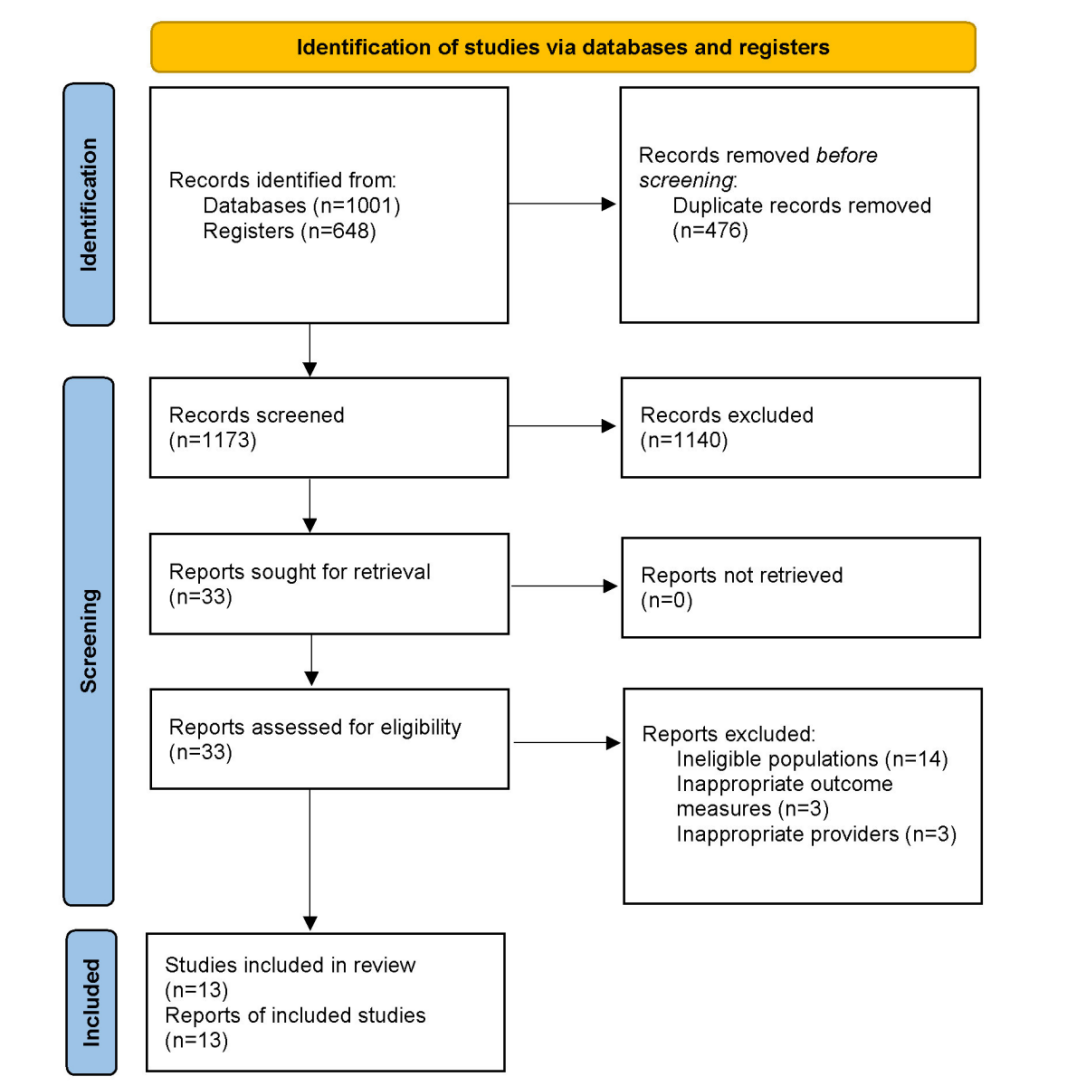
PRISMA (Preferred Reporting Items for Systematic Reviews and Meta-analyses) flow diagram.

We identified 1173 publications in our literature search after the removal of duplicates. Of these, 1140 publications were excluded based on an evaluation of the title and the brief abstract. The remaining 33 publications were assessed for eligibility, and 13 were included in our meta-analysis [24-36]. The most common reason for excluding a study was that the population studied was ineligible (n=14; Figure 1). A consensus between 2 independent reviewers was reached in 94% (31/33) of the publications.

### Quality Assessment and Publication Bias

**Figure 2:**
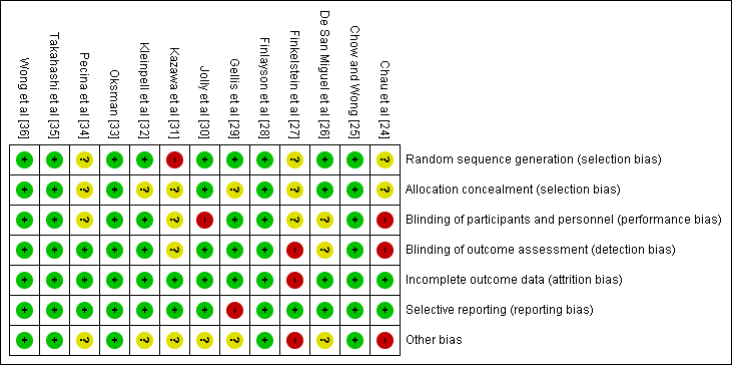
Risk of bias table.

Overall, the quality of the included RCTs was high, except in the aspects of the blinding of participants and personnel, and allocation concealment (Figure 2). Two studies were deemed to be of poor methodological quality [27,31], 2 of fair quality [26,32], and the remainder of high quality [24,25,28-30,33-36]. However, 5 studies [26,29-32] were deemed to be at an unclear risk of additional biases, through possible failures in randomization, no mention of baseline differences, and concerns over the power of the study. A summary of the risks of bias of included studies is shown in Table 1.

**Table 1:** Risk of bias in the included studies.

Study	Random sequence generation	Allocation concealment	Blinding of participants and personnel	Blinding of outcome assessment	Incomplete outcome data	Selective reporting	Other biases
Chau et al [24]	Unclear	Unclear	High	High	Low	Low	High
Chow and Wong [25]	Low	Low	Low	Low	Low	Low	Low
De San Miguel et al [26]	Low	Low	Unclear	Unclear	Low	Low	Unclear
Finkelstein et al [27]	Unclear	Unclear	Unclear	High	High	Low	High
Finlayson et al [28]	Low	Low	Low	Low	Low	Low	Low
Gellis et al [29]	Low	Unclear	Low	Low	Low	High	Unclear
Jolly et al [30]	Low	Low	High	Low	Low	Low	Unclear
Kazawa et al [31]	High	Unclear	Unclear	Unclear	Low	Low	Unclear
Kleinpell et al [32]	Low	Unclear	Low	Low	Low	Low	Unclear
Oksman et al [33]	Low	Low	Low	Low	Low	Low	Low
Pecina et al [34]	Unclear	Unclear	High	Unclear	Low	Low	High
Takahashi et al [35]	Low	Low	Low	Low	Low	Low	Low
Wong et al [36]	Low	Low	Low	Low	Low	Low	Low

### Characteristics of the Studies and Participants

Among the 13 publications, 4097 older adults were included in the meta-analysis, with 2096 older adults in intervention groups and 2001 older adults serving as controls [24-36]. The mean age of the entire sample was 73.2 (SD 4.5) years and females made up 68% (2669/3925) of the samples. The telecommunication tools that were adopted in these studies included telephones [25,28,30,33,36], home telemonitoring devices [24,26,27,29,32,34,35], and videoconferencing software or apps [27,31,35]. A total of 3 studies had nurse case managers providing telehealth services to the participants [25,29,36], another 3 studies had advanced practice nurses delivering the intervention [28,32,34], while the remainder involved registered nurses or community nurses [24,26,27,30,31,33,35]. The duration of the interventions varied from 4 weeks to 48 weeks, with a median of 24 weeks. The characteristics of the included studies are summarized in Table 2.

**Table 2:** Characteristics of the included studies.

Study	Country	Number of participants	Inclusion/exclusion criteria	Intervention components	Control group	Providers	Duration	Outcome measures	Results
Chau et al [24]	Hong Kong	N=40 (I^a^: 22, C^b^: 18)	Inclusion: aged 60 and older, with moderate or severe COPD^c^ according to the classification of the Global Initiative of Obstructive Lung Disease, admitted to hospital at least once for exacerbation during the previous year. Exclusion: unable to communicate, had impaired cognitive function, illiterate, had hearing problems, or unable to operate the telecare device.	Home visits with education on self-care and symptom management techniques; a device kit (a specially designed mobile phone, a respiratory rate sensor, and a pulse oximeter), which is used for participants’ self-monitoring of oxygen saturation, pulse rate, and respiration rate	Only home visits with education on self-care and symptom management techniques	Community nurse	8 weeks	Hospital admissions	OR^d^ 2.33 (95% CI 0.51 to 10.78)
Chow and Wong [25]	Hong Kong	N=281 (I: 96, C: 185)	Inclusion: aged 65 and older; admitted with a medical diagnosis related to chronic respiratory, cardiac, type 2 diabetes mellitus, or renal diseases; able to speak Cantonese and to communicate; resident in the hospital service area; and able to be contacted by telephone after discharge. Exclusion: identiﬁed as having cognitive problems, Mini-Mental State Examination score of <20; discharged to institutional care; followed by a designated disease management program after discharge; unable to communicate; and terminally ill.	Telephone calls, comprehensive assessment based on the OMAHA system, analysis of self-care barriers, development of mutual self-care goals, evaluation of interventions	Home visits, social calls	Nurse case managers, senior year nursing students	4 weeks	Physical component of QoL^e^ Mental component of QoL Self-efficacy	SMD^f^ 0.23 (95% CI –0.01 to 0.48) SMD 0.03 (95% CI –0.22 to 0.56) SMD 0.17 (95% CI –0.07 to 0.42)
De San Miguel et al [26]	Australia	N=71 (I: 36, C: 35)	Inclusion: Silver Chain clients with a diagnosis of COPD, receiving domiciliary oxygen, able to speak English, living in the metropolitan area. Exclusion: diagnosed with dementia, receiving palliative care, did not have a telephone landline, unable to use the telehealth equipment because of cognitive or physical impairment.	Telehealth equipment (HealthHUB), daily measurements, recording and monitoring of vital signs, assessment of general state of health, home visits, educational book about COPD, telehealth instruction manual, telephone calls, provision of support/advice/recommendations	Home visits, education book about COPD	Telehealth nurse	24 weeks	Hospital admissions	OR 0.28 (95% CI 0.10 to 0.76)
Finkelstein et al [27]	United States	N=84 (I: 40, C: 44)	Inclusion: aged 60 and older, managing 1 or more chronic diseases, not receiving Medicare home health beneﬁts but had functional limitations, able to manipulate a computer keyboard or a mouse, and had a broadband connection available in their area. Exclusion: not mentioned.	Home telehealth program using the VALUE workstation, videoconferences, electronic messages, ordering of health-related and home care services, access to health-related information, general access to the internet, physiological monitoring devices	Usual care	Telehealth nurse	8.5 weeks	Hospital admissions	OR 0.41 (95% CI 0.15 to 1.14)
Finlayson et al [28]	Australia	N=222 (I: 111, C: 111)	Inclusion: aged 65 and older; admitted with a medical condition; had at least one risk factor for readmission (aged 75 or older, admitted to a hospital more than once in the previous 6 months, multiple comorbidities, living alone, poor social support, poor self-rating of health, functional impairment, or a history of depression). Exclusion: requires home oxygen, dependent on a wheelchair or unable to walk independently for 3 m, lives in a nursing home, presence of a cognitive deficit or progressive neurological disease.	Tailored exercise program, in-home visits, telephone follow-ups, reinforcement and further explanation of the exercise program, advice and support to the caregiver	Usual care, exercise program without regular telephone follow-ups	Advanced practiced nurse, exercise physiologist	24 weeks	Hospital admissions	OR 0.40 (95% CI 0.17 to 0.92)
Gellis et al [29]	United States	N=94 (I: 48, C: 46)	Inclusion: aged 65 and older, diagnosed with heart failure or COPD, experienced frequent health care encounters (ie, hospitalized twice in the last 6 months or seen at least twice in the emergency room in the past 2 months), required 3 or more home visits per week, consented to participate in the program with random assignment, expressed a willingness to learn how to use the telehealth monitoring system. Exclusion: unable to learn to use the HomMED telehealth device due to physical disability, cognitively impaired based on a medical chart diagnosis and had no caregiver, exhibited behavioral/problems (eg, aggression, agitation, delirium, paranoia) that interfered with learning how to use the HomMED telehealth device and communicating with the telehealth nurse.	The Honeywell “HomMed” Health Monitoring System for daily monitoring of weight, noninvasive blood pressure, pulse, oxygen saturation, and temperature; further evaluation of abnormal readings by telehealth nurse, education and counseling on disease, self-care activities, and symptom management strategies	Usual care, education	Homecare telehealth nurse manager, registered homecare nurses	12 weeks	Mental component of QoL Depression	SMD 0.45 (95% CI 0.04 to 0.86)^g^ SMD –1.10 (95% CI –1.53 to –0.66)^g^
Jolly et al [30]	UK	N=516 (I: 239, C: 277)	Inclusion: has respiratory symptoms consistent with COPD, reported mild dyspnea at the baseline assessment, had a forced expiratory volume in 1 second/forced vital capacity score of <0.7 after postbronchodilator spirometry (consistent with current UK guidelines) at the baseline assessment. Exclusion: considered by doctors to be inappropriate for inclusion (eg, for having a terminal disease or a severe psychiatric disorder)	Telephone health coaching with supporting written documents, a pedometer, and a self-monitoring diary	Usual care with a standard information leaflet about the self-management of COPD	Nurse	24 weeks	QoL Self-efficacy Depression	SMD 0.18 (95% CI 0.00 to 0.36)^g^ SMD 0.23 (95% CI 0.06 to 0.41)^g^ SMD –0.15 (95% CI –0.33 to 0.03)
Kazawa et al [31]	Japan	N=32 (I: 17, C: 15)	Inclusion: had a proteinuria level of ≥2+ or a proteinuria level of 1+ and a hemoglobin A1c level of ≥7.0% (or a fasting blood sugar level of ≥130 mg/dL) at a health check conducted in 2013, and diagnosed with type 2 diabetes mellitus. Exclusion: has type 1 diabetes mellitus or gestational diabetes, had initiated dialysis, scheduled for renal transplantation in the near future, undergoing treatment for cancer, has a terminal illness, has cognitive impairment, or has a mental disorder.	Distance interviews via a tablet with a featured app (delivered to the participants by postal mail), a guidebook, a self-monitoring notebook, and foot care monofilament	Direct face-to-face interviews and intermittent telephone calls	Nurse trained in disease management	24 weeks	Systolic blood pressure (mmHg) Diastolic blood pressure (mmHg) BMI QoL Self-efficacy	SMD 6.50 (95% CI –1.44 to 14.44) SMD 0.80 (95% CI –4.02 to 5.62) SMD 3.50 (95% CI 0.55 to 6.45)^g^ SMD 0.68 (95% CI –0.15 to 1.51) SMD –0.42 (95% CI –1.23 to 0.39)
Kleinpell et al [32]	United States	N=206 (I: 134, C: 72)	Inclusion: aged ≥65 at high risk for postoperative complications; documented history of congestive heart failure; New York Heart Association functional classification of III or IV; ejection fraction of ≤40%; a history of atrial fibrillation; postdischarge complications of myocardial infarction, arrhythmias requiring treatment, reoperation, cardiac arrest, wound dehiscence, a positive wound culture; ICU^h^ stay of >2 days, mechanical ventilation for >2 days; or failure to meet clinical pathway discharge goals by postoperative day 5. Exclusion: not mentioned.	Home telemonitoring twice daily of vital signs including heart rate, blood pressure, and pulse oximetry, and daily monitoring of weight, focused reinforcement of the discharge plan	No intervention	Advanced practice nurse	4 weeks	QoL Hospital admissions	SMD –0.13 (95% CI –0.14 to 0.16) OR 0.70 (95% CI 0.32 to 1.54)
Oksman et al [33]	Finland	N=1570 (I: 970, C: 470)	Inclusion: has a glycated hemoglobin (hemoglobin A1c) level of >7, or a total cholesterol level of >4.5, or a low-density lipoprotein level of >2.3 for the previous 6 months, identified by a research nurse as being eligible for coaching. Exclusion: classified as ineligible by primary care physician, unable to co-operate or participate in health coaching, major elective surgery planned within 6 months, history of major surgery within the past 2 years, life expectancy <1 year, pregnancy.	Individual health coaching by telephone, in addition to routine social and health care, including 8 key recommendations developed by Pfizer Health Solutions: (1) know how and when to call for help, (2) learn about the condition and set goals, (3) take medicines correctly, (4) get recommended tests and services, (5) act to keep the condition well, (6) make lifestyle changes and reduce risk, (7) build on strengths and overcome obstacles, and (8) follow-up with specialists and appointments	Routine social and health care	Certified nurses and public health nurses	48 weeks	QoL	SMD 0.12 (95% CI 0.01 to 0.23)^g^
Pecina et al [34]	United States	N=166 (I: 77, C: 89)	Inclusion: aged 60 years and older with an ERA^i^ score in the highest decile. The ERA score is a composite score of previous hospitalizations, age, race, and presence of chronic disease. A high ERA score indicates an increased risk of hospitalization and emergency department visits. Exclusion: unable or unwilling to use the monitoring equipment, or if there was a concern about undiagnosed dementia after a mental status test.	Telemonitoring of biometric data using an Intel Health Guide device, questionnaires on symptoms, videoconference visits	Usual care	Geriatric nurse practitioner	48 weeks	QoL Physical component of QoL Mental component of QoL Depression	SMD 0.11 (95% CI –0.20 to 0.41) SMD –0.35 (95% CI –0.65 to –0.04) SMD –0.02 (95% CI –0.33 to 0.28) SMD 0.00 (95% CI –0.31 to 0.31)
Takahashi et al [35]	United States	N=205 (I: 102, C: 103)	Inclusion: aged ≥60; enrolled in the Employee and Community Health program primary care panel and whose ERA score exceeded 15. The ERA is an electronic database used to assess patient risk for hospitalizations or emergency department visits based on administrative data on age, sex, previous hospitalizations, and comorbid conditions (stroke, dementia, heart disease, diabetes mellitus, and chronic obstructive pulmonary disease). Exclusion: lives in a nursing home, has a clinical diagnosis of dementia or scored 29 or less in the short test of mental status, unable to use the telemonitoring system (ie, because of visual impairment or an inability to use the device).	Telemonitoring device (Intel Health Guide; Intel-GE) with real-time videoconferencing capability and peripheral measures (scales, blood pressure cuff, glucometer, pulse oximeter, and peak flow data)	Usual care	Registered nurse	48 weeks	Hospital admissions	OR 0.93 (95% CI 0.06 to 14.94)
Wong et al [36]	Hong Kong	N=610 (I: 204, C: 406)	Inclusion: admitted with a primary diagnosis related to a respiratory, diabetic, cardiac, or renal condition; Mini-Mental State Examination score of >20; able to speak Cantonese; lives within the service area; can be contacted by phone. Exclusion: discharged to an assisted care facility, being followed up by an immediate designated disease management program after discharge, unable to communicate, discharged for end-of-life care.	Telephone calls, comprehensive assessment based on the OMAHA system, develop mutual self-care goals, evaluate interventions	Home visits, placebo calls (ie, social calls)	Nurse case managers, trained nursing students	4 weeks	Self-efficacy Hospital admissions	SMD 0.19 (95% CI 0.02 to 0.36)^g^ OR 0.84 (95% CI 0.56 to 1.26)

^a^I: intervention group.

^b^C: control group.

^c^COPD: chronic obstructive pulmonary disease.

^d^OR: odds ratio.

^e^QoL: quality of life.

^f^SMD: standardized mean difference.

^g^Statistically significant.

^h^ICU: intensive care unit.

^i^ERA: Elderly Risk Assessment.

### Quantitative Synthesis

#### Quality of Life

##### Overview

A total of 5 of the 13 (38%) studies were RCTs that compared the effects of a nurse-led telehealth self-care promotion program with the usual care on the QoL of community-dwelling older adults [30-34]. The pooled SMD in the overall score for QoL was significantly different (SMD 0.12; 95% CI 0.03 to 0.20; *P*=.006; *I*^2^=21%), with the participants in the intervention group having a better QoL than those in the control group.

##### Physical Component of Quality of Life

Two studies assessed the physical component of QoL by using the Medical Outcomes Study Short Form Survey [25,34]. Pooled analyses showed that a telehealth self-care promotion program did not lead to an improvement in physical component of QoL over the usual care (SMD 0.01; 95% CI –0.18 to 0.20; *P*=.93), with high heterogeneity (χ_1_^2^=8.42; *I*^2^=88%; *P*=.004).

##### Mental Component of Quality of Life

**Figure 3:**
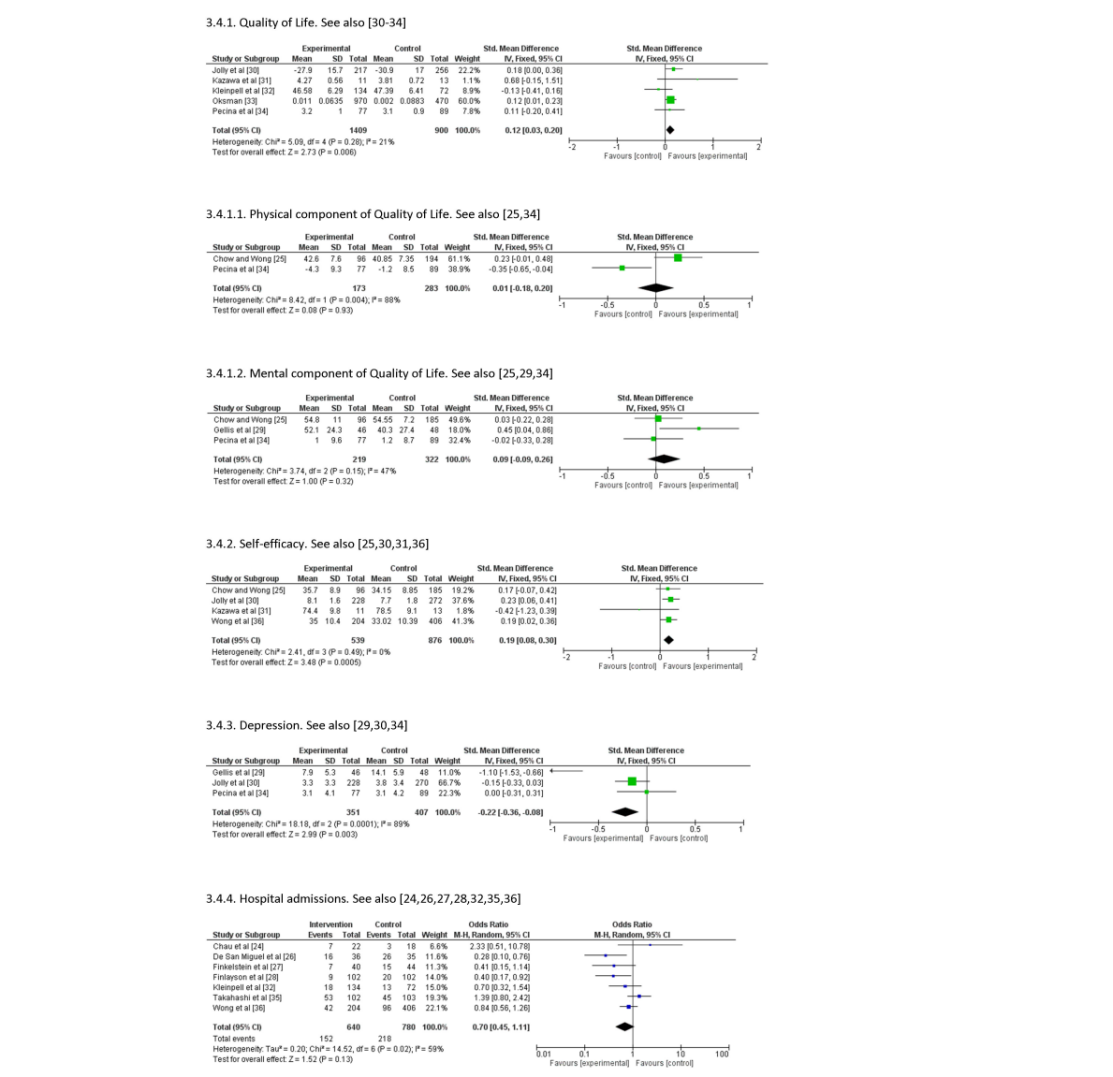
Forest plots showing the effects of nurse-led telehealth self-care promotion programmes on different outcomes.

As shown in Figure 3, the telehealth self-care promotion program did not significantly improve the mental component of QoL when compared with the usual care in the 3 studies (SMD 0.09; 95% CI –0.09 to 0.26; *P*=.32) [25,29,34]. The *I*^2^ statistics reflected moderate heterogeneity among the studies (χ_2_^2^=3.74; *I*^2^=47%; *P*=.15).

None of these outcomes showed evidence of publication bias as revealed by a visual inspection of funnel plots or the *P*-values of the Egger test (*P*>.05).

#### Self-efficacy

Four studies assessed self-efficacy [25,30,31,36], of which 2 found that the telehealth self-care promotion program had a significantly beneficial effect over the usual face-to-face care [30,36]. The pooled SMD in the overall score for self-efficacy was significantly different (SMD 0.19; 95% CI 0.08 to 0.30; *P*<.001). No evidence of heterogeneity (χ_3_^2^=2.41; *I*^2^=0%; *P*=.49) was found and there was no sign of publication bias (*P*=.71).

#### Depression

The pooled SMD in the overall score for depression was significantly different (SMD –0.22; 95% CI –0.36 to –0.08; *P*=.003) in a meta-analysis of 3/13 studies (23%) [29,30,34]. High heterogeneity (χ_2_^2^=18.2; *I*^2^=89%; *P*=.009) was indicated, but no sign of publication bias (*P*=.50) was found.

#### Hospital Admissions

Hospital admissions were reported as the outcome in 7/13 studies (54%), with 1420 participants [24,26-28,32,35,36]. Moderate heterogeneity was found among these studies (χ_6_^2^=14.5; *I*^2^=59%; *P*=.02). The number of hospital admissions in the telehealth group was 152 out of 640 (23.8%) and in the usual face-to-face group of participants was 218 out of 780 (27.9%). No significant difference was found between the groups in the number of hospital admissions (OR 0.70, 95% CI 0.45-1.11; *P*=.13).

The forest plots of all outcomes are presented in Figure 3.

## Discussion

### Principal Findings

In this review an attempt is made to summarize the evidence to ascertain the effects of nurse-led telehealth self-care programs for community-dwelling older adults in terms of QoL, self-efficacy, levels of depression, and hospital admissions. Overall, the findings of this review suggest that nurse-led telehealth programs may improve the QoL, self-efficacy, and depression levels of community-dwelling older adults when compared with the usual face-to-face care. However, no significant differences across groups were noted in hospital admissions. Although the studies seem limited in some respects, the findings of this review offer insights into the potential effectiveness of employing assistive technologies in community-based health and social care programs and on how these technologies affect the daily life of older adults, although more studies are needed to strengthen the evidence base, particularly in the aspects of organization and delivery.

Undoubtedly, the emergence of COVID-19 has led to a great global need to restructure health and social care services across patient groups, particularly regarding innovative strategies that actively support clients and their family caregivers even at a distance [37]. For community-dwelling older adults who require continuous monitoring, professional support at a distance may be an invaluable add-on to promote self-care practices. As highlighted in this review, nurse-led programs of care may lead to improvements in QoL, self-efficacy, and depression, making it a form of professional support that merits consideration. Even in studies where statistically significant findings were not observed, improvements in health outcomes such as QoL and self-efficacy were noted [31], as well as improved self-management practices [30]. A similar pattern of results was reported among persons living with cancer [38] and type 2 diabetes mellitus [39] who received nurse-led services. Taken together, the findings seem to suggest that well-designed nurse-led services delivered by trained staff may be a promising program of care that can complement and extend existing services from the health care facility to the home/community. There is, however, a need to standardize the contents and dosages of nurse-led services tailored to varied patient groups and to test these using large-scale, well-designed RCTs to strengthen the evidence base regarding their effectiveness in improving other health outcomes. In addition, a process evaluation following implementation may clarify contextual factors that can hinder or facilitate the delivery of the nurse-led programs of care and offer greater explanatory power regarding the impact of a program.

The telehealth component of the nurse-led programs of care mainly comprised structured telephone follow-ups that played an essential role in delivering education, advocacy, and coaching/behavioral change strategies. In addition, the use of customized telehealth monitoring systems installed in the homes of participants or utilized as wearable tracking devices was noted in 7 studies [24,26,27,29,32,34,35]. Evidently, as the demand for access to health care grows along with the aging population, the real-time monitoring of various physiological parameters will become a significant component of health care. Telehealth, which represents the intersection of health and technology, offers unique opportunities to deliver personalized care. The findings of this study should enable researchers and policymakers to better understand the various technologies and their effectiveness. With this understanding, they can better advise older adults on how to improve their QoL and self-efficacy and reduce their depression using appropriate assistive technologies. Besides, governments should recognize and promote the use of new technologies and the positive impact of these technologies on society, health care, and the QoL of older adults. This is because the use of these technologies not only improves the QoL of older adults but also has a positive impact on the health care system by potentially reducing health care service utilization.

Another key finding in this review is the effect of the nurse-led telehealth services on hospital admissions, which was noted to be statistically insignificant across groups. In previous studies evaluating the effects of nurse-led programs of care, the findings regarding hospital admissions were mixed. A recent integrative review that included 9 studies concluded that there is no clear evidence that community nurse–led services for older persons reduced hospital readmissions [40]. A similar finding was reported by studies involving other patient groups such as children discharged from hospital [41] and persons with heart failure [42]. By contrast, in a nurse-led program of care that focused on delivering a 4-week self-help and empowerment program for older adults living with chronic diseases, a significantly lower admission rate was observed for the intervention group compared with the control group within 84 days of an index admission [25]. Similar findings on nurse-led interventions leading to lower readmission rates have also been reported among persons with heart failure [43,44]. Although the mixed findings may be related to the nature of the interventions, the context of their delivery, or the timeline for the endpoint outcome assessment, it is likely that the intensity of the needs of the individual patients contributed to the hospitalization rates that were observed. In addition, the limitation regarding sample size across studies might make it difficult to draw conclusions. Thus, future studies are needed to address this concern/limitation to enable stronger conclusions to be drawn.

### Limitations

This meta-analysis has a few limitations. First, moderate to high heterogeneity was identified among studies that measured depression, the physical and mental components of QoL, and hospital admissions, because only 2 or 3 studies were available on these outcomes. While these studies also varied in terms of duration, content, length of follow-up, and telecommunication tool used in the programs, it was difficult to control for these differences by conducting a sensitivity analysis or a meta-regression (because there were fewer than 10 studies). Second, this study did not exclude disease-specific or transitional self-management programs that were provided by hospital-based health care professionals. Although these programs were also intended to promote self-care and health among older adults, they emphasized disease-specific skill-based training that may have been different from that in the other included studies. Participants might also have been more aware of their health after hospitalization and more willing to adhere to the recommendations of health care professionals, which led to the deviations in the results of the meta-analysis. A subgroup analysis, however, did not reveal differences between studies that focused on older adults with a specific disease and a general older population. Third, the outcome measures chosen in this study relied on subjective reports from the participants. Future RCTs may benefit from incorporating objective measurements of self-care behavior such as frequency of exercise, BMI, and the pursuit of a healthy diet.

### Conclusions

This meta-analysis of 13 RCTs revealed that nurse-led telehealth self-care promotion programs may effectively improve quality of care and self-efficacy, and alleviate depression among community-dwelling older adults. Despite the methodological limitations of the studies, including variations in the included samples, the intervention content, and the duration across studies, these results may be crucial for policymakers and health care providers to refer to when planning and designing an effective self-care health promotion program to empower older adults to take an active role in taking care of their health, be responsive to their care needs, and eventually to stay in the community with optimal well-being through the use of telehealth.

## Abbreviations

OR: odds ratio
PRISMA: Preferred Reporting Items for Systematic Reviews and Meta-analyses
QoL: quality of life
RCT: randomized controlled trial
SMD: standardized mean difference
